# Case Report: Exploring the spectrum of A20 haploinsufficiency in children

**DOI:** 10.3389/fped.2025.1624715

**Published:** 2025-07-31

**Authors:** Sahana Jayaraman, Nayimisha Balmuri

**Affiliations:** Department of Pediatric Allergy and Immunology, Johns Hopkins University School of Medicine, Baltimore, MD, United States

**Keywords:** A20 haploinsufficiency, pediatrics, genetic mutations, clinical phenotypes, symptom-specific treatments

## Abstract

Haploinsufficiency of A20 (HA20), caused by *TNFAIP3* mutations, is a rare autoinflammatory syndrome characterized by highly variable, multisystem manifestations that often delay recognition and definitive care. The A20 protein restrains NF-κB–mediated pro-inflammatory signaling; therefore, loss-of-function variants unleash widespread inflammation that can involve virtually any organ. Fewer than 200 cases have been reported worldwide; thus, clinicians have limited phenotype-specific guidance. We describe four unrelated patients evaluated at a single tertiary center, each carrying a distinct *TNFAIP3* mutation and exhibiting a unique clinical picture ranging from mucocutaneous ulcerations and Behçet-like vasculitis to lupus-like autoimmunity and periodic fever with arthritis. Tailored therapies were selected to match the dominant inflammatory pathway and produced durable clinical remission in all cases. By linking genotype, phenotype, and therapeutic response, this case series broadens the clinical spectrum of HA20 and offers practical, phenotype-driven strategies to improve screening, diagnosis, and individualized management.

## Background

**Figure 1:**
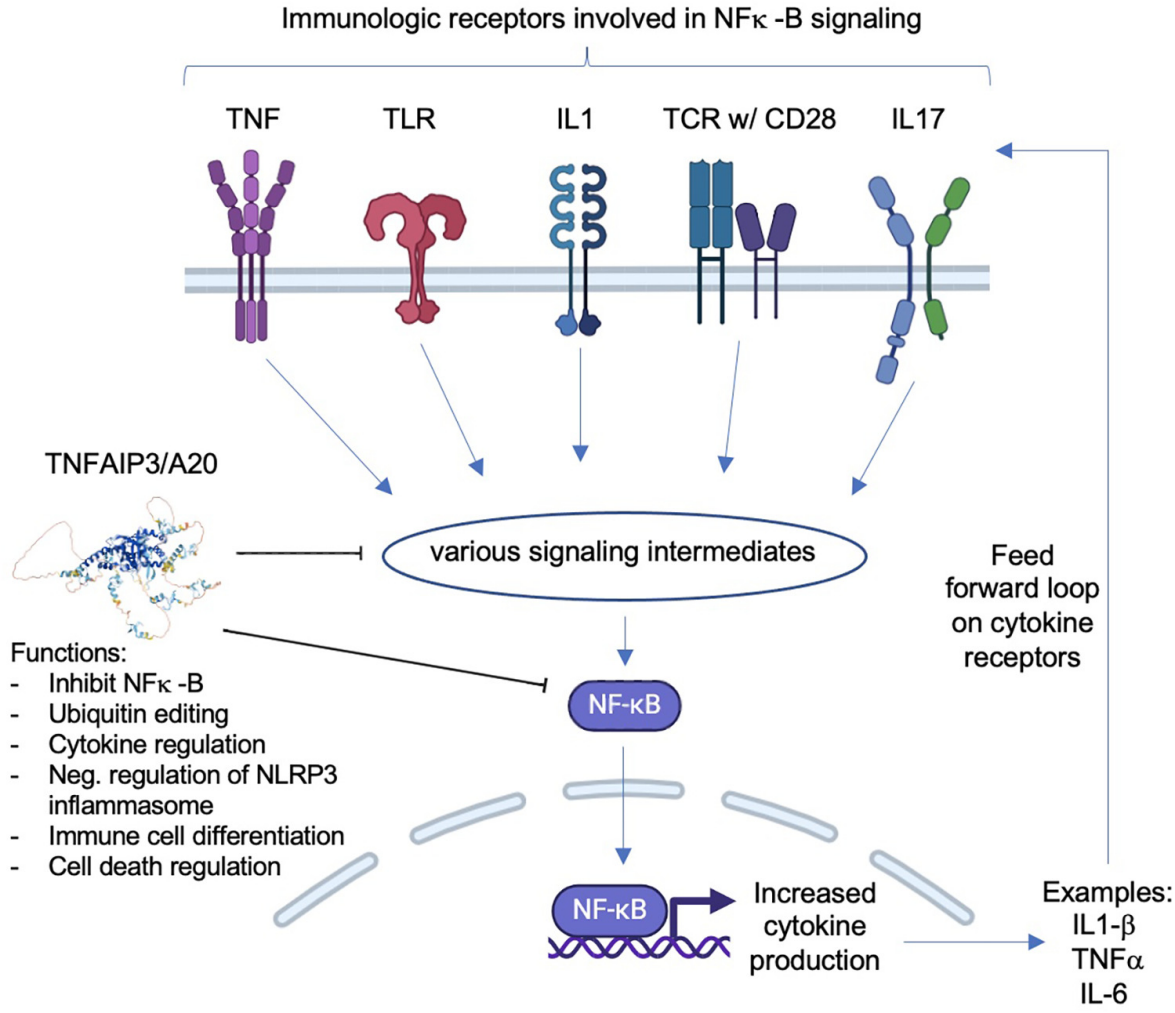
Simplified schematic of immune receptors that lead to NF-κB signaling and the role of A20 protein in this signaling. Normally, A20 can inhibit either NF-κB directly or the pathway intermediates between the activated immune receptors and NF-κB. When the inhibition is decreased due to haploinsufficiency of A20, NF-κB signaling increases, leading to increased cytokine production, which can result in a feed-forward loop where cytokine receptors that lead to NF-κB are repeatedly activated, therefore increasing downstream effects of NF-κB. Inhibitors of the shown immune receptors (which are HA20 treatment targets) prevent NF-κB signaling, thereby decreasing the pro-inflammatory effects of NF-κB.

Haploinsufficiency of A20 (HA20) is a complex immune dysregulation disorder caused by heterozygous loss-of-function mutations in the gene *TNFAIP3* (which encodes the protein TNAP3, also named A20) (1, 2). Normally, A20 acts as a negative regulator of inflammation by inhibiting NF-κB, a transcription factor which promotes inflammation in addition to contributing to many different cellular processes; thus, the loss-of-function mutations in A20 lead to a pro-inflammatory phenotype manifesting as a systemic disease that can damage multiple organs (Figure 1). While there is significant variability in clinical manifestations of the disease—variability between patient phenotypes, changes in symptoms over time in the same patient, and variability in phenotypes between family members with the same genetic mutation (the disease displays both variable expressivity and intrafamilial variability)—the following symptoms have thus far been documented as the most common (found in 30%–70% of patients): oral and genital ulcers, recurrent fevers, skin involvement (with a wide range of types of rashes), gastrointestinal disease (ranging from abdominal pain to inflammatory bowel disease phenotype), arthralgias, arthritis, and autoimmunity (3–5). Additionally, there seems to be geographic variability in disease presentation, with East Asian cohorts predominantly displaying recurrent fevers while other cohorts (mainly from the USA and Europe) predominantly develop skin rashes and ulcers (6).

Due to the variability in disease presentation and overlap in phenotypes with other autoinflammatory or autoimmune diseases (7), it can be years between initial symptoms and when the patient is correctly diagnosed. A meta-analysis reported that 43% of patients with HA20 were initially diagnosed as having Behcet's disease, followed by 26% of patients initially diagnosed with inflammatory bowel disease (7). Another meta-analysis reports that 34% of HA20 patients have autoantibodies or an autoimmune disease, most frequently antithyroid antibodies and Hashimoto's disease or systemic lupus erythematosus (4). Oftentimes, these initial diagnoses delay HA20 genetic testing because they seem to encompass the majority of patients’ symptoms. Furthermore, if these initial diagnoses are temporarily or partially controlled with therapies targeting symptoms, there is a delay in considering alternate diagnoses (4). Even after a genetic diagnosis, the rarity of HA20, with fewer than 200 cases reported worldwide, means there is limited evidence to guide treatment decisions across its diverse clinical manifestations. Currently, the most commonly reported treatments used are corticosteroids, TNF inhibitor biologics, IL-1 inhibitors, JAK inhibitors, colchicine, non-steroidal anti-inflammatory drugs, and methotrexate (3, 4). Despite HA20 being a distinct disease from Behcet's (8), given the overlap in disease phenotype with Behcet's, Behcet's treatment algorithms may be tried, many of which begin with colchicine and/or steroids plus additional agents depending on symptoms and severity (9); recent literature suggests that TNF inhibitors and IL-1 inhibitors are also effective, especially in patients with refractory disease (10). However, more data are needed for evidence-based A20-specific disease management. Thus, further reports documenting patients’ mutations, phenotypes, and treatment regimens are important for gaining a better understanding of disease trajectory and management.

**Figure 2:**
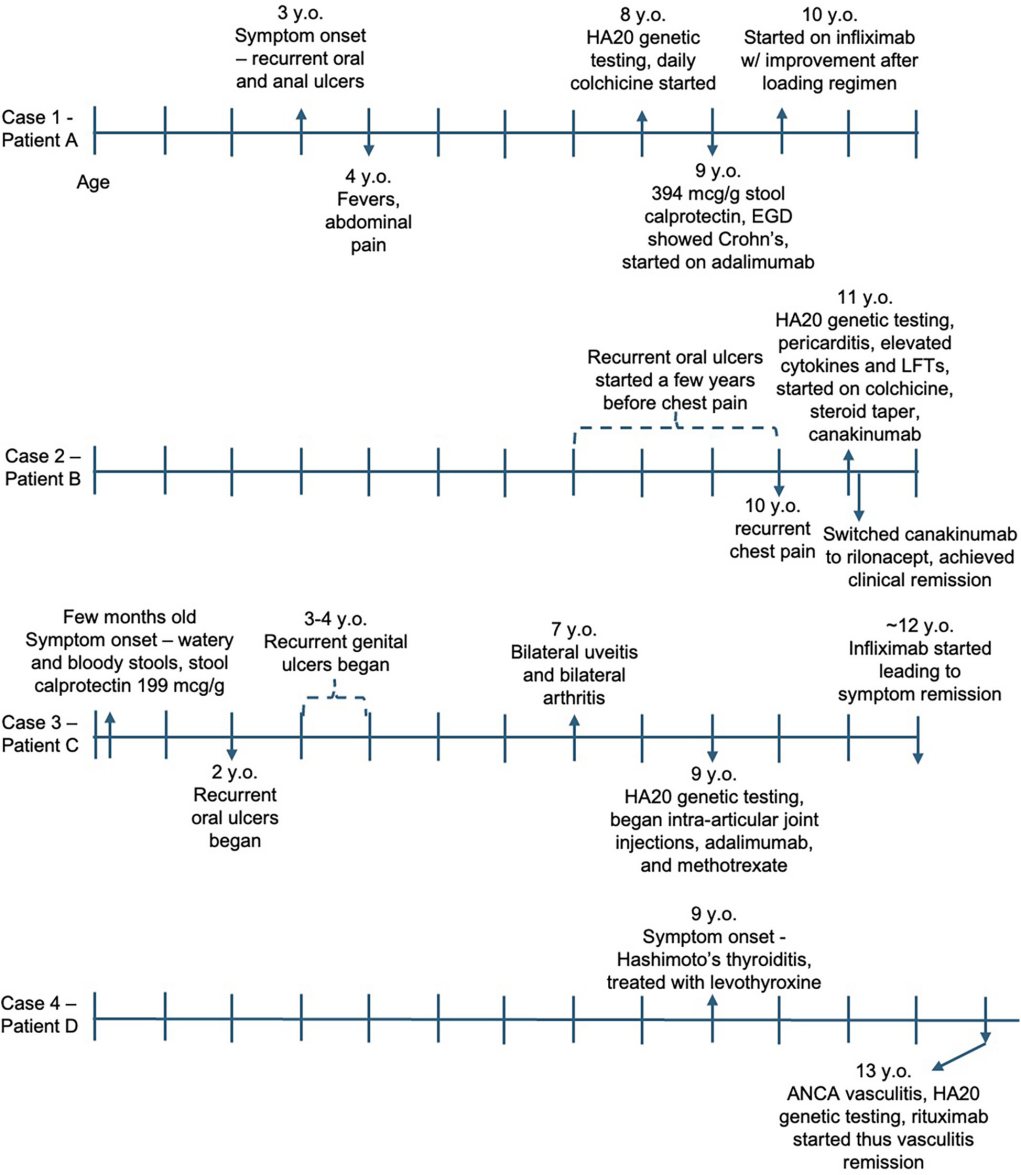
Symptom and treatment timeline for each case. Each vertical line represents 1 year, with the first line being age 0/birth. y.o., years old; LFTs, liver function tests.

This case series highlights the genetic and clinical heterogeneity of HA20 (Figure 2) and presents treatment strategies tailored to each patient’s clinical phenotype (Table 1) that resulted in successful disease outcomes.

**Table 1:** Summary table of cases and clinical phenotypes.

Patient	Age of symptom onset	Sex	Main symptoms	Treatment	Outcome
A	3 years	Female	Recurrent fevers, oral, nasal, vaginal, and anal ulcers, inflammatory bowel disease, poor appetite, and arthralgias	Infliximab and methotrexate	Resolution of all symptoms and normalized serology
B	10 years	Female	Oral ulcers and recurrent pericarditis	Rilonacept and colchicine	Resolution of all symptoms and normalized serology
C	Few months	Female	Oral and genital ulcers, arthritis, and uveitis	Infliximab and methotrexate	Resolution of all symptoms
D	9 years	Male	Hashimoto's thyroiditis, ANCA vasculitis with glomerulonephritis, nocturnal enuresis, and fatty liver	Levothyroxine for Hashimoto's and rituximab for vasculitis	Remission of Hashimoto's and vasculitis

## Case presentations

### Case 1

“A” is a 10-year-old girl with heterozygous mutation *TNFAIP3* c.740C > T (p.Pro247Leu), whose predominant symptoms include recurrent fevers, oral, nasal, vaginal, and anal ulcers, inflammatory bowel disease, poor appetite, and arthralgias. Her family history includes parental consanguinity, a paternal aunt with similar symptoms, including oral ulcers, who passed away early in life and was never formally diagnosed and a maternal aunt who develops oral ulcers and also has no formal diagnosis. She has a brother who is unaffected and a sister who has a neurodevelopmental delay. She has no family history of diagnosed autoimmune, autoinflammatory, or immunodeficiency-related diseases.

At 3 years old she developed recurrent oral and anal ulcers every 3–4 weeks. These episodes were extremely painful, leaving her unable to tolerate solid food. One year later, she developed high-grade fevers accompanied by abdominal pain and ulcers. At 8 years old, genetic testing revealed a *TNFAIP3* mutation, and her ANA was 1:160. Shortly after genetic testing, she developed increased fatigue, poor weight gain, decreased appetite, and daily abdominal pain, despite ongoing daily colchicine therapy. She also developed arthralgias (without arthritis). At the age of 9, her stool calprotectin was elevated to 394 mcg/g, ANA was 1:80, and Smith IgG was 23.1 units (although negative a year later). Due to elevated calprotectin, she underwent an esophagogastroduodenoscopy (EGD) and colonoscopy, which revealed moderate to severe Crohn's disease: gastric mucosa with mild chronic inflammation, terminal ileum with poorly formed granulomas, cecum with a poorly formed granuloma, and rectum with chronic inflammation. Screening ophthalmology visits have been negative for uveitis and retinal vasculitis.

#### Treatment course after diagnosis

At the age of 8, she was started on 0.6 mg colchicine daily uptitrated to 1.2 mg, which decreased the frequency of her ulcers and fevers. Following diagnosis of moderate to severe Crohn's disease at age 9, she was given loading doses of adalimumab per pediatric Crohn's treatment protocol (40 mg Day 1, 40 mg Day 2, 40 mg Day 15, 20 mg Day 29) and then continued on 20 mg every 2 weeks, which was increased to 20 mg weekly due to increased oral ulcers despite biweekly adalimumab. While adalimumab overall improved her weight gain and decreased the occurrence of ulcers, missed doses were associated with increased ulcers and abdominal pain and decreased appetite. She was also started on 20 mg methotrexate (initial oral preference) to prevent antidrug antibodies against adalimumab, in addition to synergistic treatment with adalimumab. However, this increased her nausea, so she was switched to subcutaneous methotrexate. Unfortunately, while her symptoms improved remarkably, she continued having fatigue, mucosal ulcers, and abdominal pain along with elevation of C-reactive protein (CRP), erythrocyte sedimentation (ESR), and stool calprotectin; thus, 1 year after using adalimumab, she was transitioned to 5 mg/kg infliximab every 8 weeks, post loading regimen, which has been shown to help in treatment-refractory disease courses (11). Clinically, she reported feeling better after the first infliximab dose, with further improvement observed on exam and laboratory measurements after completion of loading doses. Her manifestations of fatigue, ulcers, abdominal pain, and poor appetite have now resolved with normalized CRP, ESR, and stool calprotectin. Methotrexate has been decreased to 10 mg weekly for the prevention of antidrug antibodies against infliximab. She has also been transitioned off of colchicine.

### Case 2

“B” is an 11-year-old girl with a heterozygous mutation *TNFAIP3* c.503G>A (p.Trp168*), whose predominant symptoms include oral ulcers and recurrent pericarditis. She has a family history positive for HA20. Her father has HA20 of the same genetic variant [with symptoms of aphthous ulcers, gastrointestinal inflammation, diffuse large B-cell lymphoma (DLBCL) in the jejunum, atherosclerosis, large vessel vasculitis of coronaries and carotids requiring four-vessel bypass at a young age, and significant cutaneous findings]. He only required treatment in the past few years, which started with anakinra and then transitioned to the TNF-alpha blocker, adalimumab. Her paternal grandfather also had HA20 (required bypass surgery at a young age and developed thyroid cancer). Her paternal great-grandmother had joint issues, oral ulcers, and rheumatic fever but never had genetic testing. Her mother is unaffected, and one of her two brothers has many oral ulcers but is otherwise healthy. Neither brother has been genetically tested.

From ages 10 to 11, her symptoms began as three episodes of chest pain every 2–3 months for which workup in the emergency department (ED) was negative [normal chest x-ray (CXR) and electrocardiogram] aside from elevated white blood cell count and CRP/ESR. At the age of 11, she was diagnosed with HA20 by an Invitae panel, which was ordered due to her family history. A few months after diagnosis, she developed pericarditis as confirmed by echocardiogram which demonstrated mild pericardial effusion, along with CXR demonstrating small pleural effusions. At the time of her pericarditis diagnosis, cytokine panel revealed elevations of the following (in pg/ml): IL-2R of 2,500.8, IL10 of 18.6, IL17 of 2.3, and IL6 of 95.7. The absolute CD4 count was low at 363 cells/mm^3^. Immunoglobulins were elevated as follows (in mg/dl): IgG 1,400, IgA 328, and IgM 218. Liver function tests were elevated with AST of 40 U/L and ALT of 64 U/L. Stool calprotectin was mildly elevated to 148 mcg/g. Transglutaminase antibodies were elevated to 15 U/ml. She has had episodes of oral ulcers (1–2 ulcers at a time), approximately four times a year for the past several years. Endoscopy and colonoscopy showed lymphoid aggregates in the duodenum, but biopsies were without clear celiac disease or inflammatory bowel disease.

#### Treatment course after diagnosis

During her hospitalization for pericarditis, she was started on 1.2 mg colchicine, 1 mg/kg divided three times a day of indomethacin, and a steroid taper. She remained on 1.2 mg colchicine, indomethacin, and the steroid taper outpatient (2 mg/kg/dose, daily). Given her elevated IL-2R, fever with pericarditis and serositis, and family history, IL-1 blockade was started. She was initially trialed on 4 mg/kg canakinumab every 4 weeks. However, she was unable to wean off steroids (required 1 mg/kg/dose daily) due to chest pain and developed breakthrough pericarditis (clinically and on echocardiogram) with a viral infection after two doses of canakinumab, requiring a temporary increase to her oral steroids to 2 mg/kg/dose daily. She was then transitioned to recurrent pericarditis dosing of rilonacept (one time loading dose of 4.4 mg/kg subcutaneously followed by a maintenance dose of 2.2 mg/kg weekly), which resolved her clinical symptoms after one dose. She was weaned off prednisone and indomethacin completely after four doses, and her cytokine panel and stool calprotectin normalized after 3 months of therapy.

### Case 3

“C” is a 12-year-old girl with a heterozygous mutation *TNFAIP3* c.811C>T (p.Arg271*) (HLA B51 negative) whose predominant symptoms include oral and genital ulcers, arthritis, and uveitis. Her maternal aunt died of lupus in her 20s, but otherwise she has no family history of autoimmune, autoinflammatory, or immunodeficiency-related diseases. Her two sisters are unaffected.

At a few months old, she had episodes of watery and bloody stools with a stool calprotectin of 199 mcg/g, and at 2 years old, she began having recurrent oral ulcers. Around 3–4 years old, she began having recurrent genital ulcers. At the age of 7, she developed bilateral anterior uveitis and bilateral arthritis of her knees with a leg length discrepancy. At the age of 9, the Invitae panel identified her *TNFAIP3* genetic mutation.

#### Treatment course after diagnosis

She received intra-articular joint injections and began treatment with 20 mg adalimumab, which was increased to 40 mg every 2 weeks rather than 20 mg weekly to help with medication compliance. Oral methotrexate 10 mg was also added to aid in synergy and prevent antidrug antibodies against adalimumab. She was initially improving on adalimumab, but due to complex social dynamics in the household, follow-up with appointments and medication compliance were inconsistent; thus, C had multiple flares (oral ulcers, genital ulcers, and active arthritis). She was transitioned to infliximab 5 mg/kg/dose every 8 weeks, post loading regimen. After 4 months of this regimen, she developed breakthrough oral ulcers and arthritis; thus, infliximab was uptitrated to 7.5 mg/kg/dose every 6 weeks. Since this change, she has remained consistent with medications, including methotrexate. She has had resolution of arthritis, uveitis, and mucosal ulcers. Her abdominal pain has resolved, and her appetite has normalized.

### Case 4

“D” is a 14-year-old boy with a *de novo* heterozygous mutation, *TNFAIP3* c.1135C>T (p.Q379*), as both parents are negative for the mutation. His predominant symptoms are Hashimoto's thyroiditis (managed with 50 mcg levothyroxine), ANCA vasculitis with glomerulonephritis, nocturnal enuresis, and fatty liver. He has one sibling, an older brother who has anxiety and epilepsy. His father has diverticulitis, but his mother is unaffected. His family history is otherwise negative for autoimmune, autoinflammatory, or immunodeficiency-related diseases.

At 9 years old, he was diagnosed with Hashimoto's thyroiditis. At 13 years old, he presented to the ED with 3 days of generalized abdominal pain and was found to have significant hematuria, proteinuria, CRP of 60.9 mg/L, C-ANCA 1:640, anti-PR3 >160 immunofluorescence units (neg <2), and anti-MPO 0.3 immunofluorescence units (neg <3.5). The combination of Hashimoto's and ANCA positivity (11, 12) prompted whole-exome genetic testing, and he was found to have HA20. His sinus and pulmonary computed tomography (CT) scans were normal. His most recent anti-PR3 level was 2.2 immunofluorescence units, and he continues to have occasional nosebleeds but no other sinus symptoms or pain.

#### Treatment course after diagnosis

His ANCA vasculitis has been managed with two doses of rituximab induction dosing (750 mg/m^2^) and a steroid taper (currently off steroids). He will continue with rituximab every 6 months for maintenance of vasculitis remission. Unfortunately, “D” did not have a renal biopsy at diagnosis, so we are uncertain about the accuracy of the initial pathology. However, he has responded to rituximab clinically and serologically. Should his renal manifestations flare, a biopsy was strongly recommended. We have a low threshold for adding TNF inhibition based on cytokine panels, clinical status, or future biopsy results (13).

## Discussion

Here, we have presented three young female patients and one young male patient, who presented to the same tertiary care center with different HA20 disease courses and phenotypes. All patients developed initial symptoms of disease at varying ages, ranging from a few months old, to a few years old, to past the age of 10 (which is within the known range of disease onset of 1 week old to 39 years old) (14). Despite presenting only four cases here, three out of four patients developed symptoms before the age of 10, aligned with a study that identified 73% of patients (*n* = 61) display their initial symptom before 10 years old (8). Additionally, while three patients have recurrent ulcers as a predominant symptom, their accompanying symptoms ranged from arthralgias, inflammatory bowel disease (both of which are common in HA20), to rarer symptoms such as autoimmune disease manifested as thyroiditis, vasculitis, uveitis, and arthritis (3, 4). Only one patient had a definite genetic predisposition with her father sharing the same mutation, but it is important to note that she developed symptoms at an earlier age than her father and with a different phenotype of symptoms thus far.

Because HA20 leads to increased inflammation through factors such as NF-κB which has widespread cellular functions, one hypothesis is that distinct HA20 mutations would lead to distinct disease phenotypes (as demonstrated by these four patients) based on the nuances of the downstream effects of those mutations. Three out of four patients had non-sense mutations in line with previously reported meta-analyses where the majority of patients had non-sense mutations (4, 14, 15). Furthermore, three out of four patients had mutations in the ovarian tumor unit (OTU), and one patient had a mutation in the region between the OTU domain and the zinc finger (ZF) 1 domain. It has been previously reported that 56% of patients had mutations that affected both OTU and ZF domains (14), in line with our study, as two patients had non-sense mutations in the OTU region and one patient had a non-sense mutation in the region between OTU and ZF. Only one patient in this report has a missense mutation in the OTU region.

The most commonly reported symptoms of HA20 (oral and genital ulcers, recurrent fevers, skin involvement, and gastrointestinal disease) are found in all mutation groups, with variability in certain mutation types being more or less associated with joint involvement and autoimmune disease (4, 14). Loss-of-function non-sense mutations may be more associated with bipolar aphthous ulcers, gastrointestinal disease, and autoimmunity, and all three of our patients with non-sense mutations have symptoms that fall into one or more of these categories (4). Missense mutation p.Pro247Leu in patient A has been previously reported in a male patient (our patient is female) with similar symptoms as follows: early onset at age 4 with recurrent fevers, abdominal and joint pains, and inflammatory bowel disease upon endoscopy, ultimately treated with infliximab and thalidomide (15, 16). While there are only a few reported cases with this mutation, similar symptoms could suggest a specific genotype–phenotype correlation as more patients are diagnosed. Mutation p.Arg271* has previously been reported in several patients and identified as pathogenic (17–21); however, a few previously identified patients are male, and while many patients have oral and genital ulcers, only one other patient (female) reports oral ulcers and uveitis (2, 12, 22). Mutation p.Trp168* has been identified as pathogenic (ClinVar RCV005102078.1) (23) and identified in a patient with both HA20 and DLBCL, as found in the father of patient B (24). As pericarditis is extremely rare in HA20 reported cases, further patient data are needed to determine potential genotype–phenotype correlations. Mutation p.Q379* has been identified as pathogenic (ClinVar RCV005052457.1) (23) and has been reported in patients with primary breast marginal zone lymphomas (25) and an HA20 patient (also male) whose primary symptoms were fever, anal ulcerations, skin abscesses, and abdominal pain with a family history including a mother and grandmother who were diagnosed with Behcet's (26). As vasculitis is rare in HA20 reported cases, further patient data are needed to determine potential genotype–phenotype correlations. It is also important to note that these mutations are unlikely to be the sole contributors of distinct phenotypes, as one patient and her father share a mutation but have different disease trajectories, and some of our reported genotypes have different phenotypes compared with those reported in the literature.

In addition to the noted similarities and differences between this case series and previously published cohorts, it is important to mention this report's limitations. As we present only four cases here, this small sample size and retrospective discussion prevent us from correlating genotypes and phenotypes, as well as correlating the success of treatment regimens with multiple patients of the same phenotype. Additionally, we have limited longitudinal data and are unable to comment on the length of symptom remission for patients given these specific regimens. Despite these limitations, we hope these cases will provide a better starting point for physicians considering HA20 screening and diagnosis, as well as treatment strategies specific for phenotypes. Additionally, these cases will provide data about genotype–phenotype pairs that future research can build on to generate correlations.

Given the widespread impact of HA20 on cellular pathways and variability of disease phenotypes, HA20 is important to have in the differential diagnoses of patients with recurrent ulcers, a known family history, or two or more autoimmune diseases. Delayed diagnosis leads to a delay in a successful treatment regimen, which can prolong patient and family suffering, as well as cause patients to be subjected to excessive lab testing when a unifying diagnosis is unclear but symptoms recur and/or evolve. Earlier genetic testing, especially when recurrent symptoms are reported, can provide patients and families with a diagnosis earlier and thus lead to quicker treatment initiation and symptomatic improvement.

Additionally, with current treatment strategies based on other diseases, all four patients displayed improvement in their symptoms. Cases 1 and 3 (Patients A and C) were started on anti-TNF therapy, which is the most commonly used treatment type for HA20 patients with GI involvement (4). While they were both started on adalimumab, Patient A required transition to infliximab given persistent abdominal pain, mucosal ulcers, and continued elevation of ESR, CRP, and stool calprotectin, and Patient C required transition to infliximab as it improved medication compliance. In both cases, infliximab led to symptom resolution. Case 2 (Patient B) was started on colchicine and steroids with the addition of IL-1 blockade, all of which are also common treatments for patients with HA20 (3). Given her elevated IL-2R, fever with pericarditis and serositis, and family history of successful treatment with IL-1 blockade, she was started on canakinumab, but due to breakthrough pericarditis, she was transitioned to rilonacept as it is specific for recurrent pericarditis, which led to symptom resolution. Case 4 (Patient D) is treated with levothyroxine for Hashimoto's and rituximab for his ANCA vasculitis, both of which have kept corresponding symptoms in remission. Given that most of our patients required trial and transition to different therapies, regular disease monitoring is important, as is continuing to adjust treatments until symptom resolution and inflammatory laboratory normalization.

Expanding data on HA20, including patient presentations, disease trajectories, and treatment responses, is essential for improving screening and advancing our understanding of genotype and phenotype correlations and effective disease management.

## Data Availability

The original contributions presented in the study are included in the article/Supplementary Material, further inquiries can be directed to the corresponding author.
